# Dopamine is involved in reparative dentin formation through odontoblastic differentiation of dental pulp stem cells

**DOI:** 10.1038/s41598-023-32126-1

**Published:** 2023-04-06

**Authors:** Shoko Fujino, Sayuri Hamano, Atsushi Tomokiyo, Risa Sugiura, Daiki Yamashita, Daigaku Hasegawa, Hideki Sugii, Shinsuke Fujii, Tomohiro Itoyama, Hirofumi Miyaji, Hidefumi Maeda

**Affiliations:** 1 Department of Endodontology and Operative Dentistry, Faculty of Dental Science, Kyushu University, 3-1-1 Maidashi Higashi-ku, Fukuoka, 812-8582 Japan; 2 Oral Health/Brain Health/Total Health Research Center, Faculty of Dental Science, Kyushu University, 3-1-1 Maidashi Higashi-ku, Fukuoka, 812-8582 Japan; 3 Department of Endodontology, Kyushu University Hospital, 3-1-1 Maidashi Higashi-ku, Fukuoka, 812-8582 Japan; 4 Laboratory of Oral Pathology, Division of Maxillofacial Diagnostic and Surgical Sciences, Faculty of Dental Science, Kyushu University, 3-1-1 Maidashi Higashi-ku, Fukuoka, 812-8582 Japan; 5 Department of Periodontology and Endodontology, Faculty of Dental Medicine, Hokkaido University, 7 Kita13-jonishi Kita-ku, Sapporo, 060-8586 Japan

**Keywords:** Health care, Dentistry, Dental materials, Dental treatments, Endodontics

## Abstract

Conventional direct pulp-capping materials induce pulp cells to secrete various biomolecules in pulp tissues that promote reparative dentin formation through induction of odontoblastic differentiation of dental pulp stem cells (DPSCs). However, these biomolecules sometimes induce bone-like dentin with poor sealing properties. Therefore, exploration of biomolecules that allow tight sealing by tubular reparative dentin is required. We recently reported that dopamine (DA) is involved in dentinogenesis. Hence, we investigated the effect of DA on odontoblastic differentiation of DPSCs and reparative dentin formation. Both tyrosine hydroxylase (TH), a DA synthetase, and DA were expressed in odontoblast-like cells in vivo. In vitro, their expression was increased during odontoblastic differentiation of DPSCs. Furthermore, TH-overexpressing DPSCs had promoted odontoblastic differentiation and DA production. Moreover, DA stimulation promoted their differentiation and induced tubular reparative dentin. These results suggest that DA produced by TH is involved in odontoblastic differentiation of DPSCs and has an inductive capacity for reparative dentin formation similar to primary dentin. This study may lead to the development of therapy to preserve vital pulp tissues.

## Introduction

When deep caries or trauma cause irreversible pulp exposure, pulpectomy is frequently performed. However, in non-vital teeth, tooth fracture and progressive caries tend to occur compared with vital teeth, which sometimes lead to teeth loss^1–3^. Therefore, pulp conservation is very important for preserving teeth.

Direct pulp capping is a useful treatment to preserve vital pulp tissues in cases of pulp exposure. This treatment promotes reparative dentin formation by the application of calcium-based direct pulp-capping materials to the exposed pulp. Previous reports have observed cells expressing odontoblastic markers such as Dentin matrix acidic phosphoprotein 1 (Dmp-1), Dentin sialophosphoprotein (Dspp), and Nestin, beneath the reparative dentin after direct pulp capping^4–6^. These cells are regarded as odontoblast-like cells. Odontoblast-like cells are considered to different from dental pulp stem cells (DPSCs) and are associated with reparative dentin formation^7^.

Calcium-based direct pulp-capping materials, such as calcium hydroxide (CH) and mineral trioxide aggregate (MTA) cement, are often used for direct pulp capping. These materials induce pulp cells to secrete various biomolecules that promote odontoblastic differentiation of DPSCs, which induce reparative dentin formation^8^. However, CH has high solubility and poor sealing ability^9^, and MTA has a long setting time and poor handling properties^10^. Moreover, it has been reported that the long-term success rate of this treatment is only 56–81%^11^. Therefore, to increase the success rate, many studies have been conducted to develop new evidence-based pulp-capping materials that enhance the odontoblastic differentiation of DPSCs. Bone morphogenetic protein-4, fibroblast growth factors, and insulin-like growth factor-1 promote odontoblastic differentiation of DPSCs^12,13^. However, these biomolecules often form bone-like dentin that does not have the structure of dentinal tubules unlike primary dentin. Bone-like dentin has poor sealing properties and carries a risk of reinfection with bacteria because the tissue contains porosity and tunnel defects^14^. Therefore, the exploration of biomolecules that induce reparative dentin with a dentinal tubular structure similar to that of primary dentin is required.

Tyrosine hydroxylase (TH) is the rate-limiting enzyme in the biosynthesis of dopamine (DA), a monoamine neurotransmitter. The alteration of TH expression regulates DA expression in various tissues^15,16^. DA plays an important role in motion control and immunity regulation^17^, and is also involved in bone formation. Recent reports have shown that DA promotes osteoblastic differentiation of bone marrow stem cells and MC3T3-E1 cells^18,19^. Furthermore, in our previous report, we revealed that the involvement of DA in the maturation of odontoblasts at the stage of tooth development^20^. However, there is no report about the effect of DA on odontoblast-like differentiation of DPSCs in the process of reparative dentin formation.

Therefore, we aimed to determine whether DA promotes odontoblastic differentiation of DPSCs and reparative dentin formation with the structure of dentinal tubules similar to primary dentin.

## Results

### Th and DA expression in odontoblast-like cells in the rat direct pulp-capping model

**Figure 1:**
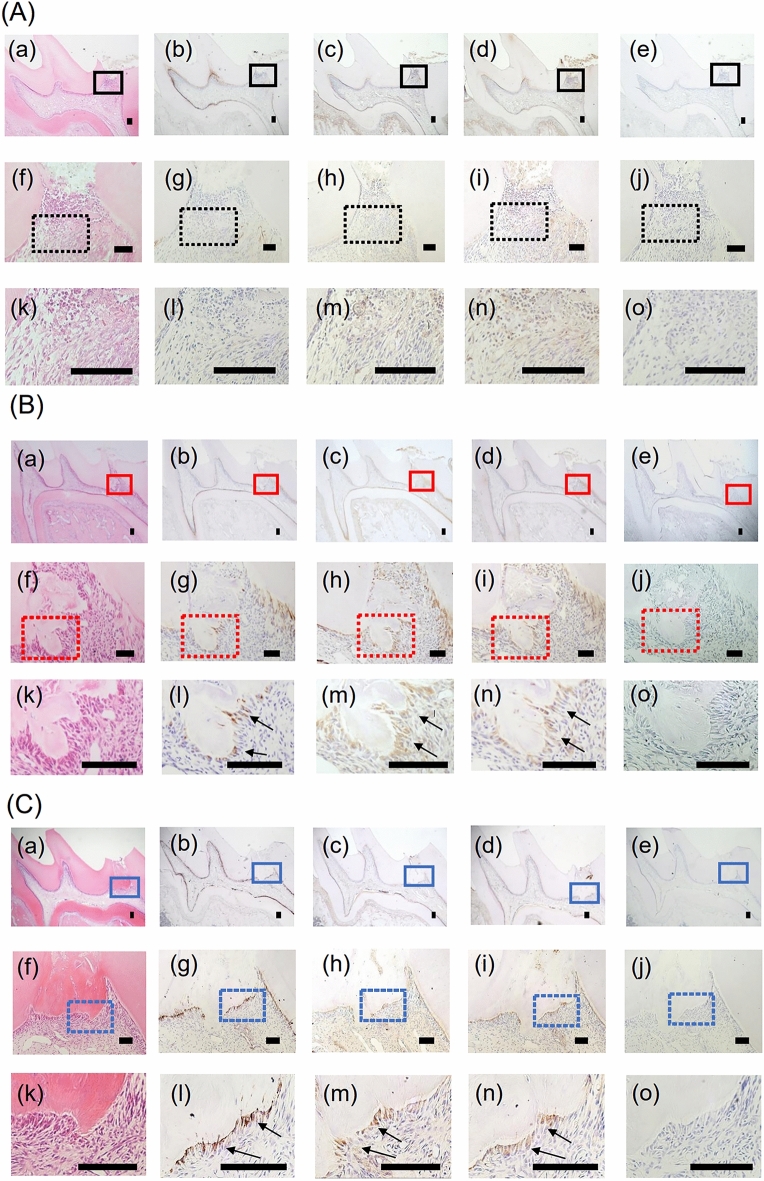
Expression of Th and DA in odontoblast-like cells in rat direct pulp capping model. Images of maxillary first molars at day 3 [**A**(**a**–**o**)], day 7 [**B**(**a**–**o**)], and day 21 [**C**(**a**–**o**)] after direct pulp capping treatment with MTA cement. HE staining of the maxillary first molars [**A**(**a**, **f**, **k**)], [**B**(**a**, **f**, **k**)], [**C**(**a**, **f**, **k**)]. Immunohistochemical staining of the maxillary first molars with anti-Th antibody [**A**(**b**, **g**, **l**), **B**(**b**, **g**, **l**), **C**(**b**, **g**, **l**)], anti-DA antibody [**A**(**c**, **h**, **m**), **B**(**c**, **h**, **m**), **C**(**c**, **h**, **m**)], and anti-Nestin antibody [**A**(**d**, **i**, **n**), **B**(**d**, **i**, **n**), **C**(**d**, **i**, **n**)]. [**A**(**f**–**j**)] Magnified images of the black boxed areas in [**A**(**a**–**e**)]. [**A**(**k**–**o**)] Magnified images of the black dotted boxed areas in [**A**(**f**–**j**)]. [**B**(**f**–**j**)] Magnified images of the red boxed areas in [**B**(**a**–**e**)]. [**B**(**k**–**o**)] Magnified images of the red dotted boxed areas in [**B**(**f**–**j**)]. [**C**(**f**–**j**)] Magnified images of the blue boxed areas in [**C**(**a**–**e**)]. [**C**(**k**–**o**)] Magnified images of the blue dotted boxed areas in [**C**(**f**–**j**)]. [**A**(**e**, **j**, **o**), **B**(**e**, **j**, **o**), **C**(**e**, **j**, **o**)] Rabbit control IgG was used as a negative control. Nuclei were stained with hematoxylin. Bars = 100 μm. Arrows; odontoblast-like cells.

**Figure 2:**
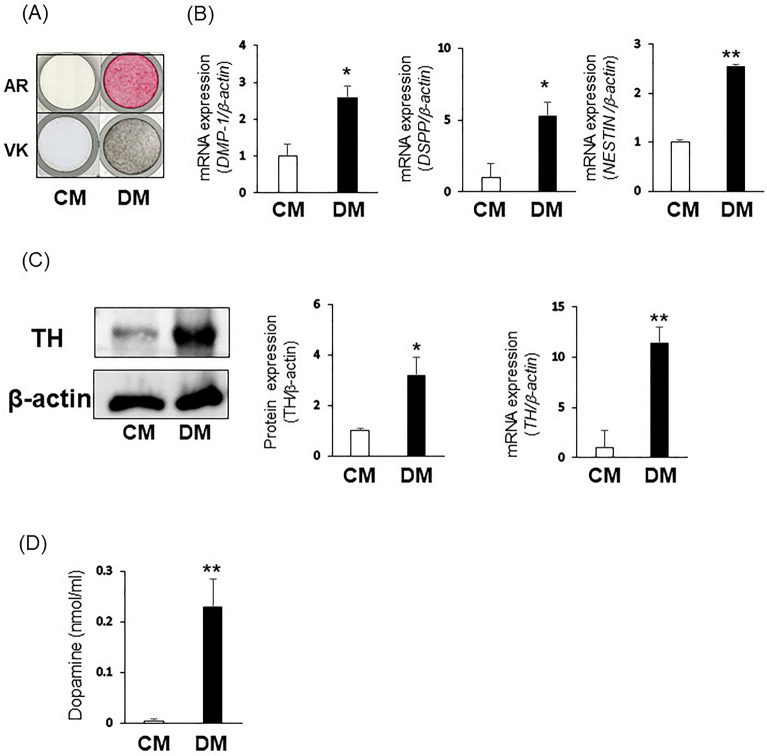
TH and DA expression during odontoblastic differentiation of DPSCs-3U. (**A**–**D**) 3U-DPSCs were cultured in CM or DM. (**A**) After 7 days of cultivation, calcium deposits were analyzed by Alizarin Red S staining and von Kossa staining. (**B**) After 3 days of cultivation, gene expression of DMP-1, DSPP, NESTIN was examined by quantitative RT-PCR. (**C**) Gene and protein expression of TH was investigated by quantitative RT-PCR and Western Blotting. The bands were quantified using ImageJ. Original blots are presented in Supplementary Information 8. (**D**) DA concentration of the culture supernatant of DPSCs was analyzed by ELISA. β-actin was used an internal control. Statistical analysis was performed using the Student’s unpaired t-test with Easy R software. n = 3, **P < 0.01, *P < 0.05.

To investigate the expression of Th and DA in odontoblast-like cells, we performed immunohistochemical staining of Th, DA, and Nestin in the maxillary first molar using the rat direct pulp-capping model. Nestin was used as an odontoblastic differentiation marker. At 3 days after direct pulp capping, reparative dentin was not found [Fig. 1A(a, f, k)] and positive cells for anti-Th [Fig. 1A(b, g, l)], DA [Fig. 1A(c, h, m)], and Nestin [Fig. 1A(d, i, n)] antibodies were not detected under the pulp exposure site. At 7 days after treatment, reparative dentine was partially generated on the edge of the exposed pulp [Fig. 1B(a, f, k)]. Th [Fig. 1B(b, g, l)], DA [Fig. 1B(c, h, m)], and Nestin [Fig. 2B(d, i, n)] expression was observed in odontoblast-like cells beneath the reparative dentin. However, positive cells were not found at a distance from the reparative dentin. Additionally, at 21 days after treatment, reparative dentine formation was almost completed [Fig. 1C(a, f, k)] and odontoblast-like cells beneath the reparative dentin displayed intensely positive reactions for anti-Th [Fig. 1C(b, g, l)], DA [Fig. 1C(c, h, m)], and Nestin [Fig. 1C(d, i, n)] antibodies. Normal control IgG showed no positive reactions [Fig. 1A(e, j, o), B(e, j, o), C(e, j, o)].

In accordance with the above results, we hypothesized that TH and DA were involved in odontoblast-like differentiation of DPSCs. Therefore, we examined the role of TH and DA in the differentiation of DPSCs.

### TH and DA expression during odontoblastic differentiation of DPSCs

We examined the expression of mesenchymal stem cell (MSC) markers in CD146, a DPSC marker, -positive dental pulp cells and their multipotency to evaluate their stem cell characteristics. Flow cytometry showed that DPSCs expressed MSC markers, CD29, CD90, and CD105 (Sup. 1A, G). However, they did not express hematopoietic stem cell markers such as CD34 and CD45 (Sup. 1B, H). Next, we investigated the osteogenic and adipogenic differentiation potencies of DPSCs. After 4 weeks of cultivation in each differentiation medium, positive areas of Alizarin Red S (Sup. 1C, I) and Oil Red O (Sup. 1E, K) staining were observed. Gene expression of osteogenic and adipogenic differentiation markers was also increased in DPSCs cultured in each differentiation medium (Sup. 1D, F, J, L). Therefore, we used CD146-positive dental pulp cells as DPSCs in this study. DPSCs were cultured in control medium (CM) or odontoblastic differentiation medium (DM) for 7 days. Alizarin Red S and von Kossa staining demonstrated calcification in DPSCs cultured in DM (Fig. 2A and Sup. 2A). Gene expression of the odontoblastic differentiation markers *DSPP*, *DMP-1*, and *NESTIN*, in DPSCs cultured in DM was increased compared with that in DPSCs cultured in CM (Fig. 2B and Sup. 2B). Additionally, TH expression was significantly higher in DPSCs cultured in DM than that in the control [Fig. 2C (gene; CM vs. DM: 3.57E−06 ± 2.43E−06 vs. 4.06E−05 ± 5.97E−06, *P* = 0.00247, protein; CM vs. DM: 2868.23 ± 309.26 vs. 9087.81 ± 2099.31, *P* = 0.0176) and Sup. 2C]. Furthermore, we measured the DA concentration of in the supernatant of DPSCs cultured in CM or DM for 7 days using enzymE−linked immunosorbent assay (ELISA). The DA concentration of the supernatant of DPSCs cultured in DM was higher than that of DPSCs cultured in CM [Fig. 2D (CM vs. DM: 0.0032 ± 0.0043 vs. 0.229 ± 0.056, *P* = 0.00672) and Sup. 2D].

### Increased odontoblastic differentiation of TH-overexpressing DPSCs

**Figure 3:**
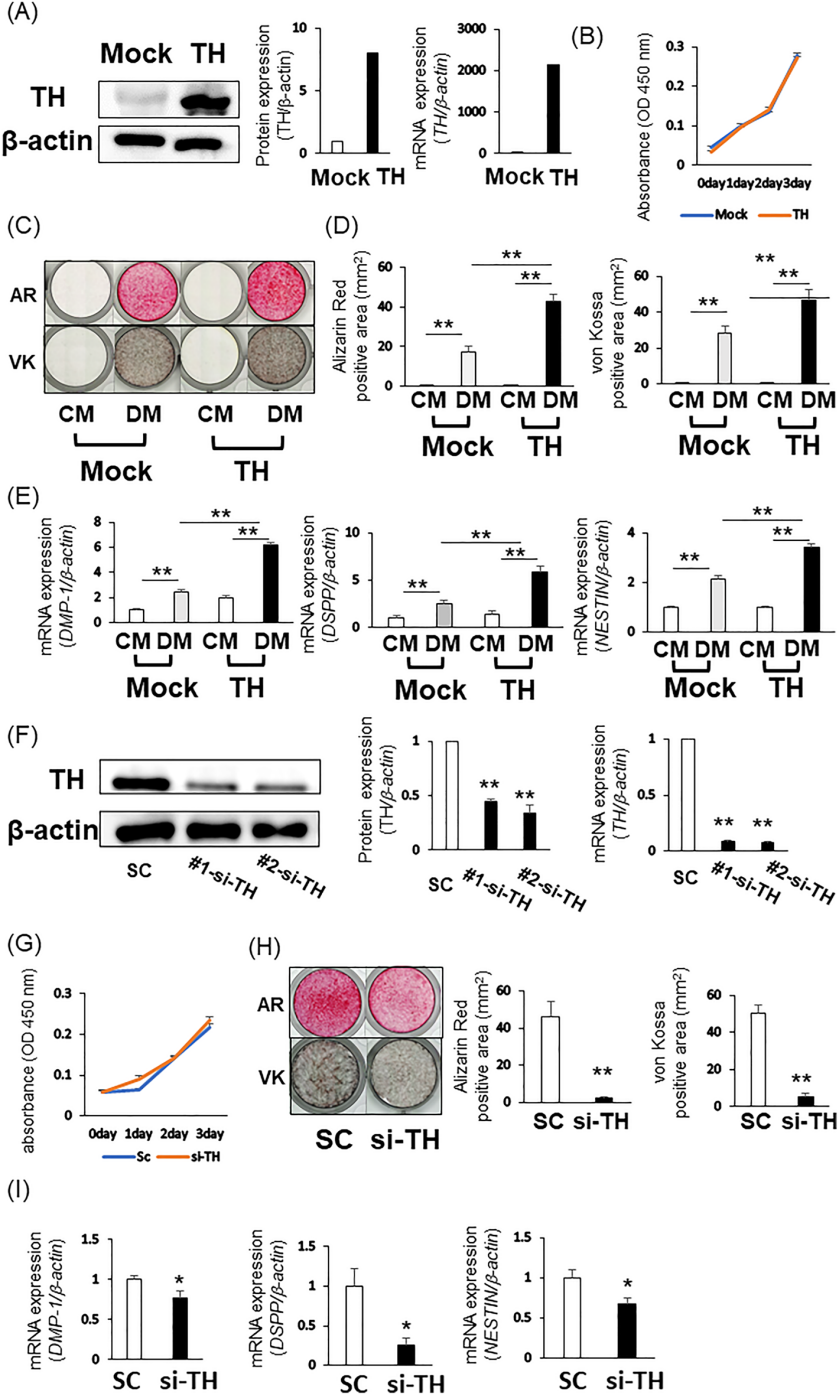
Effects of TH overexpression on the odontoblastic differentiation of DPSCs-3U. (A-E) Mock- and TH-DPSCs were cultured in CM or DM. (**A**) Gene and protein expression of TH were investigated by quantitative RT-PCR and Western blotting. The bands were quantified using ImageJ (**B**) Proliferation of Mock-DPSCs or TH-DPSCs was examined by WST-1 assay. (**C**) Alizarin Red S staining and von Kossa staining of Mock-DPSCs or TH-DPSCs cultured in CM or DM. (**D**) Positive area of Alizarin Red S staining and von Kossa staining was quantified using a Keyence BZ-9000 microscope with BZ-H4M/BZ-H4C/BZ-H4CM software. (**E**) Gene expression of DMP-1, DSPP, and NESTIN was investigated by quantitative RT-PCR. (**F**–**I**) TH-DPSCs was transfected with control siRNA or TH siRNA. (**F**) Gene and protein expression of TH were confirmed by quantitative RT-PCR and Western blotting. The bands were quantified using ImageJ. (**G**) Proliferation of TH-DPSCs transfected with control siRNA or TH siRNA was examined by WST-1 assay. (**H**) Alizarin Red S staining and von Kossa staining of TH-DPSCs transfected with control siRNA or TH siRNA cultured in DM. The positive areas were quantified by Keyence BZ-9000 microscope with BZ-H4M/BZ-H4C/BZ-H4CM software. (**I**) Gene expression of DMP-1, DSPP, and NESTIN was examined by quantitative RT-PCR. β-actin was used an internal control. Statistical analysis was performed using the Student’s unpaired t-test or one-way ANOVA followed by Bonferroni’s test with Easy R software. n = 3, **P < 0.01, *P < 0.05. Original blots are presented in Supplementary Information 8.

To assess the effect of TH overexpression on odontoblastic differentiation of DPSCs, we generated DPSCs expressing Mock or TH using lentivirus particles termed Mock-DPSCs and TH-DPSCs, respectively. The gene and protein expression of TH in TH-DPSCs was higher than that in Mock-DPSCs (Fig. 3A and Sup. 3A). There was no difference in proliferative capacity between Mock-DPSCs and TH-DPSCs (Fig. 3B and Sup. 3B). The positive areas of Alizarin Red S (Mock vs. TH: 17.17 ± 2.84 vs. 42.89 ± 3.7, *P* = 0.00005) and von Kossa (Mock vs. TH: 28.1 ± 4.16 vs. 46.77 ± 5.65, *P* = 0.00079) staining of TH-DPSCs cultured in DM were significantly larger than those of Mock-DPSCs cultured in DM (Fig. 3C, D and Sup. 3C, D). Additionally, TH overexpression in DPSCs promoted gene expression of odontoblastic differentiation markers [Fig. 3E (*DMP-1*; Mock vs. TH: 2.48E−05 ± 2.075E−06 vs. 6.24E−05 ± 1.73E−06, *P* = 0000001, *DSPP*; Mock vs. TH: 7.75E−05 ± 1.29E−05 vs. 1.84E−04 ± 1.8E−05, *P* = 000033, *NESTIN*; Mock vs. TH: 1.29E−02 ± 9.69E−04 vs. 2.08E−02 ± 8.778E−04, *P* = 0000029) and Sup. 3E].

Next, we transfected TH siRNA into TH-DPSCs. Western blotting and RT-PCR analysis demonstrated that TH expression in TH-DPSCs transfected with TH siRNA was lower than that in TH-DPSCs transfected with control siRNA (Fig. 3F and Sup. 3F). There is no difference in the proliferative capacity between TH-DPSCs transfected with control siRNA or TH-siRNA (Fig. 3G and Sup. 3G). TH knockdown in TH-DPSCs decreased mineralization and gene expression of odontoblastic differentiation markers (Fig. 3H, I and Sup. 3H, I).

### DA production in Mock-DPSCs and TH-DPSCs

**Figure 4:**
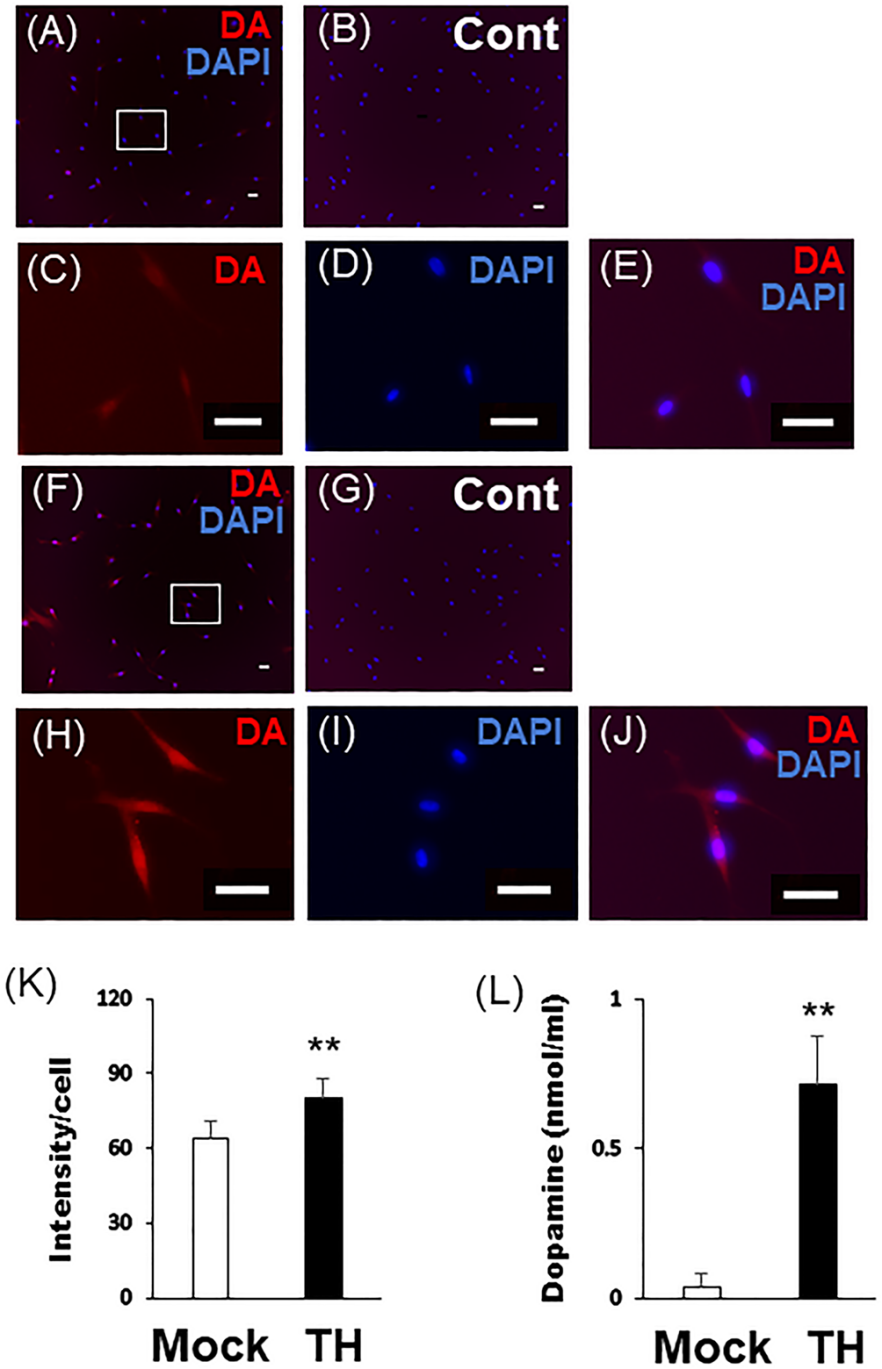
Dopamine production in Mock- or TH-DPSCs-3U. (**A**–**J**) Immunofluorescence staining of Mock-DPSCs or TH-DPSCs with anti-DA antibody (red) was performed. (**B**, **G**) Rabbit control IgG (cIgG) was used as a negative control. (**C**–**E**) Magnified images of the white boxed areas in (**A**). (**H**–**J**) Magnified images of the white boxed areas in (**F**). Nuclei were stained with DAPI. Bars = 50 μm. (**K**) Intensity of immunofluorescence staining of DA were quantified using a Keyence BZ-9000 microscope with BZ-H4M/BZ-H4C/BZ-H4CM software. (**L**) Mock-DPSCs or TH-DPSCs were cultured in DM for 30 min. The concentration of DA of each culture supernatant was measured by ELISA analysis. Statistical analysis was performed using the Student’s unpaired t-test with Easy R software. n = 3, **P < 0.01.

DA is synthesized from tyrosine via TH and increases by an increase of TH. Therefore, we examined DA production in Mock-DPSCs and TH-DPSCs by immunocytochemistry and ELISA. We found that DA in the cytoplasm [Fig. 4A–K (Mock vs. DA: 64 ± 8.6034 vs. 75 ± 6.6094, *P* = 1.45768E−06) and Sup. 4A–K] and culture supernatant [Fig. 4L (Mock vs. DA: 0.169 ± 0.0712 vs. 0.8689 ± 0.1617, *P* = 0.00711) and Sup. 4L] of TH-DPSCs was higher than that in Mock-DPSCs.

### Effects of DA on odontoblastic differentiation of DPSCs and reparative dentin formation

We examined DA receptor expression in DPSCs. All dopamine receptors (*D1R*, *D2R*, *D3R*, *D4R*, and *D5R*) were expressed in DPSCs (Sup. 5A, B).

**Figure 5:**
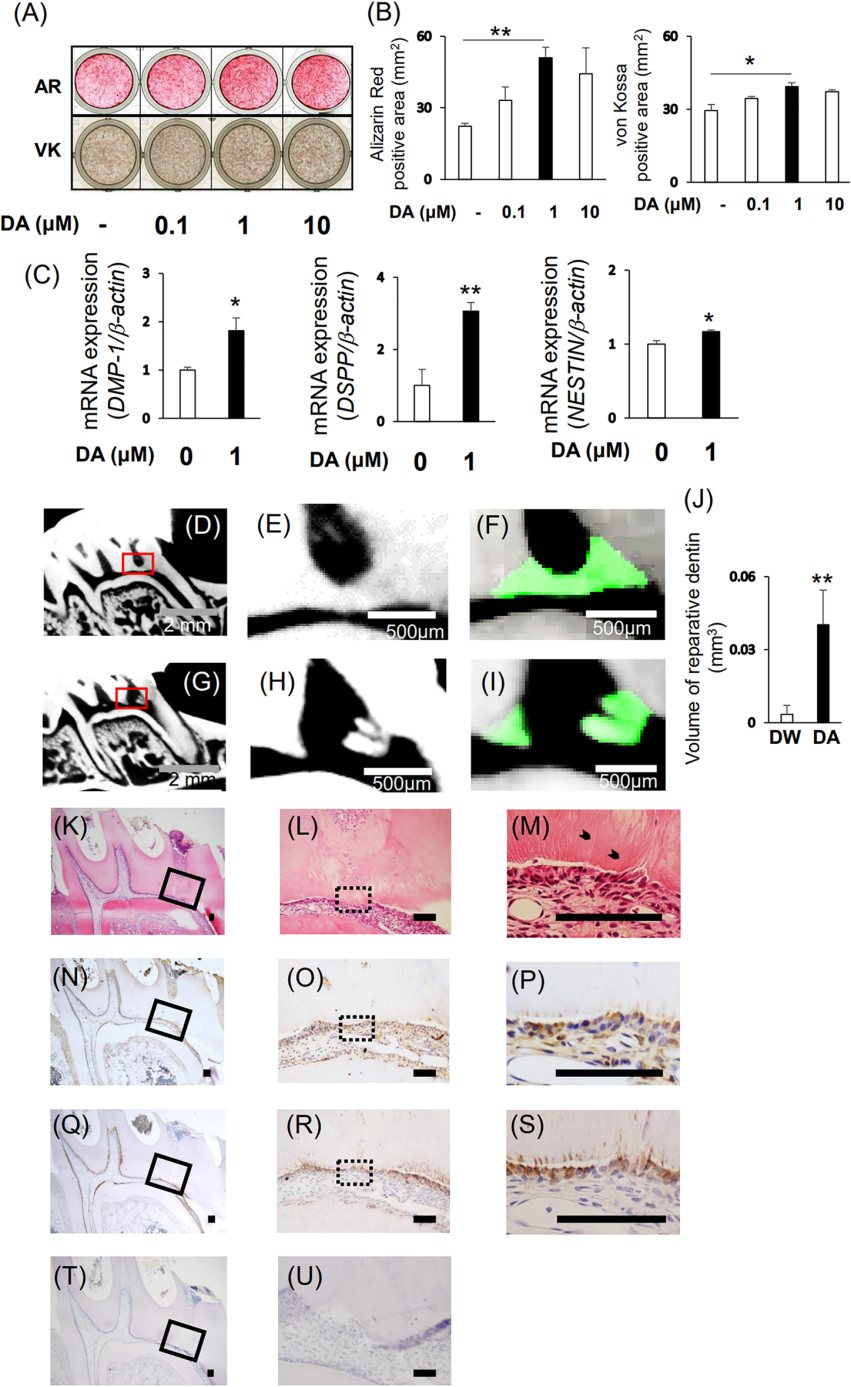
Effects of DA on the odontoblastic differentiation of DPSCs-3U and on the reparative dentin formation. (**A**–**C**) DPSCs were cultured in DM containing with or without dopamine (0.1, 1, and 10 μM). (**A**) After 7 days of cultivation, calcium deposits were analyzed by Alizarin Red S staining and von Kossa staining. (**B**) Positive area of Alizarin Red S staining and von Kossa staining were quantified using a Keyence BZ-9000 microscope with BZ-H4M/BZ-H4C/BZ-H4CM software. (**C**) After 3 days of cultivation, the gene expression of DMP-1, DSPP, and NESTIN were investigated by quantitative RT-PCR. β-actin was used an internal control. Statistical analysis was performed using the Student’s unpaired or one-way ANOVA followed by Bonferroni’s test with Easy R software. t-test. n = 3, **P < 0.01, *P < 0.05. (**D**–**U**) The surface of exposed pulp was capped with nano β-TCP/collagen scaffold containing DA (**D**–**F**, **K**–**U**) or DW (**G**–**I**). (**D**–**I**) μCT images of the maxillary first molars at day 21 after direct pulp capping treatment with DA (**D**–**F**) or DW (**G**–**I**). (**E**, **F**) Magnified images of the red boxed areas in (**D**). (**H**, **I**) Magnified images of the red boxed areas in (**G**). (**F**, **I**) Newly reparative dentin was displayed in green. (J) The reparative dentin volume of these images was quantified using TRI/3D/VIE-FCS software. (**K**–**M**) HE staining of the maxillary first molars in DA treatment group. (**K**–**U**) Immunohistochemical staining of the maxillary first molars in DA treatment group with anti-Nestin antibody (**N**–**P**) and anti- Th antibody (**Q**–**S**). (**L**, **O**, **R**) Magnified images of the black boxed area in (**K**, **N**, **Q**). (**M**, **P**, **S**) Magnified images of the dotted black boxed area in (**L**, **O**, **R**). (**T**, **U**) Rabbit control IgG was used as a negative control. (**U**) Magnified images of the black area in (**T**). Nuclei were stained with hematoxylin. Bars = 100 µm. Arrowheads; dentinal tubules. Statistical analysis was performed using the Student’s unpaired test with Easy R software. t-test. n = 6, **P < 0.01.

To examine the effect of DA on odontoblastic differentiation of DPSCs, we cultured DPSCs in DM containing with DA (0, 0.1, 1, and 10 μM). Alizarin Red S- (DM vs, 1 μM DA: 22.3 ± 1.13 vs. 51 ± 4.47, *P* = 0.00014) and von Kossa- (DM vs. 1 μM DA: 29.47 ± 2.55 vs. 39.38 ± 1.52, *P* = 0.031) positive areas of 1 μM DA-treated DPSCs were the greatest of all groups (Fig. 5A, B and Sup. 6A, B). Similarly, the expression of odontoblastic differentiation markers was also significantly increased in the presence of 1 μM DA [Fig. 5C (*DMP-1*; DM vs. DA: 1.04E−05 ± 6.36222E−07 vs. 1.88E−05 ± 2.75556E−06, *P* = 0.0172, *DSPP*; DM vs. DA: 1.94E−05 ± 8.57556E−06 vs. 5.94E−05 ± 4.59E−06, P = 0.00556, *NESTIN*; DM vs. DA: 1.23E−02 ± 5.97778E−04 vs. 1.44E−02 ± 2. 9 E−04, *P* = 0.015) and Sup. 6C].

To elucidate the effect of DA on reparative dentin formation, we used DA as a direct pulp-capping material. The surface of the exposed pulp was capped with a nano β- tricalcium phosphate (TCP)/collagen scaffold containing 10^–2^, 10^–1^, and 1 M DA (DA group) or demineralized water (DW [Control group]). At 21 days after surgery, reparative dentin was not found in the 10^–2^ M DA group (Sup. 7A, B). Incomplete reparative dentin was observed in the 10^–1^ M DA group (Sup. 7C, D), and reparative dentin formation was promoted in the 1 M DA group. Therefore, we used 1 M DA concentration in this study (Fig. 5K–M). Moreover, the volume of reparative dentin formation was significantly larger in the DA group than that in the Control group (Fig. 5D–J, Control vs. DA: 0.0034 ± 0.00372474 vs. 0.040346207 ± 0.014094676, *P* = 0.00049). The DA group exhibited hard tissue that completely sealed the surface of the exposed pulp and displayed the structure of dentinal tubules (Fig. 5K–M), while the Control group showed defective hard tissue with no dentinal tubular structure (Sup. 8A–C).

Next, to confirm whether cells beneath the hard tissue induced by DA were odontoblast-like cells, we performed immunohistochemical staining of Nestin and Th. Nestin [Fig. 5L–N and Sup. 9A–C(d, e)] and Th (Fig. 5O–Q) were expressed in cells beneath the reparative dentin. Furthermore, positive reactions for anti-Nestin (Fig. 5N–P) and anti-Th (Fig. 5Q–S) antibodies were observed in dentinal tubules. However, the cells beneath the hard tissues in the Control group had a faint positive reaction to their antibodies [Sup. 8D–I and Sup. 9D–F(d, e)]. Normal control IgG showed no positive reactions (Fig. 5T, U, J, K).

Finally, to assess whether DPSCs had gathered beneath the exposed pulp capped with the nano β-TCP/collagen scaffold containing DA or DW, we performed immunohistochemical staining of CD146. At 3 days after treatment, a small number of CD146-positive cells were founded beneath the exposed pulp both in the DA group [Sup. 9A(b, c)] and the Control group [Sup. 9D(b, c)]. At 7 days after treatment, the number of CD146-positive cells beneath the exposed pulp had increased in both groups [Sup. 9B(b, c), E(b, c)]. Additionally, at 21 days after treatment, CD146-positive cells were observed beneath the hard tissues [Sup. 9C(b, c), F(b, c)].

## Discussion

A direct pulp-capping model treated with MTA was used to observe odontoblastic differentiation of DPSCs during reparative dentin formation in vivo. Odontoblast-like cells beneath the reparative dentin express odontoblastic markers. Among them, Nestin is expressed in both odontoblasts and odontoblast-like cells, and its expression level increases as odontoblastic differentiation progresses in pre-odontoblasts and DPSCs^21,22^. Therefore, we used Nestin to confirm the presence of odontoblast-like cells. In this study, cells expressing Nestin were found beneath the reparative dentin as observed in previous reports. Moreover, TH and DA expression were confirmed in these cells. TH is a DA synthesis enzyme and is known as a sympathetic nerve marker. TH and DA are expressed in nerve tissues and are involved in the differentiation of nerve cells^23^. Moreover, these factors are expressed in non-neural cells such as those in the heart and pancreas^24,25^. Even in osteoblasts and pre-odontoblasts, these factors are expressed and promote their differentiation^20,26^. Taken together, we demonstrated that TH and DA were involved in odontoblastic differentiation of DPSCs during reparative dentin formation.

We also investigated the effect of TH and DA on odontoblastic differentiation of DPSCs in vitro. MTA releases calcium ions into pulp tissues, which increases the expression of various factors involved in odontoblastic differentiation of DPSCs, such as bone morphogenetic protein-2 and transforming growth factor beta-1^27,28^. Moreover, our previous study showed that CaCl_2_ stimulation induces their odontoblastic differentiation through calcium-sensing receptors^29^. In this study, CaCl_2_-treated DPSCs had increased gene expression of odontoblastic markers as well as TH and DA compared with untreated DPSCs. Therefore, TH and DA were associated with odontoblastic differentiation of DPSCs similarly to the in vivo environment.

Previous reports have revealed that TH overexpression increases DA not only in nerve cells, but also in non-neural cells such as lymphocytes and cardiac progenitor cells. Moreover, DA secreted from these cells promotes their differentiation in an autocrine or paracrine manner^24,30^. DA released from sympathetic nerves promotes osteoblastic differentiation of MC3T3-E1 cells and bone marrow stem cells through dopamine receptors^18,31–34^. Baudry et al.^35^ reported that in pulp tissues, DA released from planets was important in the recruitment of DPSCs for the reparative dentin formation after pulp injury. In this study, TH-overexpressing DPSCs had increased DA in their cytoplasm and culture supernatants. Additionally, TH overexpression and DA treatment promoted their odontoblastic differentiation. Therefore, TH upregulates DA production in DPSCs, which might be associated with their odontoblastic differentiation in an autocrine or paracrine manner.

Next, we examined the effect of DA on reparative dentin formation in vivo. Previous studies have reported that reparative dentin has the structure of dentinal tubules and is associated with odontoblast-like cells expressing odontoblastic markers, while bone-like dentin does not have this structure and odontoblast-like cells are rarely found beneath bone-like dentin^4^. Our results showed that DA induced hard tissues with the structure of dentinal tubules, and moreover, both Nestin- and Th- positive cells were observed beneath the hard tissues induced by DA, similarly to reparative dentin induced by MTA. However, in the Control group, the hard tissues with no dentinal tubular structure were detected, and the cells beneath the hard tissues had a faint positive reaction to anti- Nestin and TH antibodies. Therefore, these results suggest that DA has the capacity to induce reparative dentin formation with the structure of dentinal tubules.

Nano β-TCP/collagen scaffolds were used in this study. Our previous studies have reported that the nano β-TCP/collagen scaffolds containing with various proteins in direct pulp capping model promote reparative dentin formation^36,37^. The concentration of proteins added to the scaffolds in vivo was higher than that of proteins stimulating cells in vitro. Therefore, we observed the formation of reparative dentin with concentrations of 10^−2^ M, 10^–1^ M, and 1 M DA. The results showed that reparative dentin was not found in the 10^–2^ M DA group. Incomplete reparative dentin was observed in the 10^–1^ M DA group, and reparative dentin formation was promoted in the 1 M DA group. Based on these results, we set 1 M DA concentration in this study. However, the reparative dentin induced by 1 M DA had a little porosity. To improve this, we assumed that we should increase the DA concentration, although previous reports showed that high DA concentrations cause cytotoxicity^38^. There was a limit to the use of higher DA concentrations. Komichi et al., have reported that they used gelatin as a scaffold for direct pulp capping in vivo^39^. Therefore, future research should investigate more suitable scaffolds that can be added to a lower concentration of DA to create a new direct pulp-capping material.

DPSCs are thought to be recruited from pulp tissues and differentiate into odontoblast-like cells during reparative dentin formation^7^. Cells expressing stem cell markers have been observed around exposed pulp during reparative dentin formation^39^. Similar to previous reports, we found that cells positive for CD146-, a DPSC marker, had gathered around exposed pulp tissues in both the DA group and the Control group. Moreover, Nestin- positive cells around the exposed pulp during reparative dentin formation induced by DA were detected, while the cells around exposed tissues in the Control group had slightly positive staining. Therefore, DA might promote reparative dentin formation through the induction of odontoblastic differentiation of DPSCs.

In this study, we found that Th and DA were expressed in odontoblast-like cells beneath reparative dentin, and that TH overexpression promoted odontoblastic differentiation of DPSCs and the production of DA. Additionally, DA induced odontoblastic differentiation of DPSC and reparative dentin formation with dentinal tubules similar to those of primary dentin. Hence, DA produced by TH was involved in odontoblastic differentiation of DPSCs and reparative dentin formation. This study may lead to the development of a therapy to preserve vital pulp tissues.

## Methods

### Isolation of dental pulp stem cells

Human pulp cells were extracted from healthy premolars of 25-year-old female (3U) and 19-year-old male (5C) who visited Kyushu University hospital for orthodontic treatment, with informed consent as described previously^29^. We used CD146-positive pulp cells as DPSCs because CD146 is thought to be a specific DPSC markers^40,41^. DPSCs were isolated from human pulp cells using anti-CD146 magnetic microbeads and MACS® Magnetic separators (Miltenyi Biotec Inc., CA, USA) following the manufacturer’s protocol. All procedures were carried out following the rules of the Declaration of Helsinki and in accordance with the requirements of the Kyushu University Certified Institutional Review Board for Clinical Trials. The study was approved by Kyushu University Certified Institutional Review Board for Clinical Trials (approval number: 2–115).

### Adipogenic differentiation of DPSCs

DPSCs (2 × 10^4^ cells/well on 24-well plates) were cultured in 10% fetal bovine serum (FBS; Sigma-Aldrich; St. Louis, MO, USA)/alpha-minimum essential medium (α-MEM; Gibco-BRL, Grand Island, NY, USA) supplemented with 0.5 mM methylisobutylmethylxanthine (Sigma-Aldrich), 0.5 mM hydrocortisone (Sigma-Aldrich), and 60 µM indomethacin (Sigma-Aldrich) for 1 mouth. After cultivation, intracellular fat droplets were detected by Oil Red O (Sigma-Aldrich) staining.

### Osteogenic differentiation of DPSCs

DPSCs (2 × 10^4^ cells/well on 24-well plates) were cultured in 10% FBS/α-MEM containing with 50 μg/mL l-ascorbic acid phosphate (FUJIFILM Wako Pure Chemical Corporation, Osaka, Japan), 1 × 10^−7^ M dexamethasone (Merck Millipore, Darmstadt, Germany), and 2 mM glycerol 2-phosphate (FUJIFILM Wako). After 4 weeks of cultivation, mineralized deposits were stained by Alizarin Red S.

### Odontoblastic differentiation of DPSCs

DPSCs were seeded on 24-well plates (2 × 10^4^ cells/well) and cultured in α-MEM containing 10% FBS and 2 mM CaCl_2_ as odontoblastic differentiation medium (DM) in accordance with our previous study^29,42,43^. After 7 days of cultivation, mineralized deposits were stained by Alizarin Red S and von Kossa, and quantified under a BZ-9000 microscope (Keyence, Osaka, Japan) with BZ-H4M/BZ-H4C/BZ-H4CM software (Keyence, version 1.12.4). Additionally, the gene expression of odontoblastic differentiation markers (*DSPP*, *DMP-1*, and *NESTIN*) was investigated by quantitative real-time reverse transcription-PCR (RT-PCR) analysis.

### Rat direct pulp-capping model

Seven- or eight-week-old male Wistar rats (Kyudo, Saga, Japan) were used for the direct pulp-capping model as described previously^37^. Briefly, rats were anesthetized using intraperitoneal anesthesia composed of 2 mg/kg midazolam (Sandoz, Tokyo, Japan), 0.15 mg/kg medetomidine hydrochloride (Kyoritsuseiyaku, Tokyo, Japan), and 2.5 mg/kg butorphanol tartrate (Meiji Seika Pharma, Tokyo, Japan). The mesial angle part of upper first molars were shaved with a no. 1/2 round steel bur until it was close to the pulp under a microscope. The size of a cavity (1 mm in diameter and 0.5 mm in depth) was approximately measured using a periodontal probe. DA (0.189 g) was dissolved in 1 ml of DW. The DA solution (1 μl) or DW (1 μl) was added to nano β-TCP/collagen scaffolds (L 1 mm × W 1 mm × H 0.5 mm) with good tissue compatibility and the ability to retain proteins^37,44^. Scaffolds containing DW were used as a control. After an exploratory needle was used to expose the pulp, the surface of the exposed pulp was capped with MTA cement (Dentsply Sirona; Charlotte, NC, USA) or the prepared scaffolds. The cavity was sealed with glass ionomer cement (GC Fuji IX EXRA; GC, Tokyo, Japan) which exhibits good adhesion to dentin even under wet conditions^45^. This study was approved by the ethics committee of the Kyushu University Animal Experimental Center (protocol number; A22-288-0). All procedures were carried out following the relevant guidelines and regulations of the Kyushu University, and the study was performed in accordance with ARRIVE (Animal Research: Reporting of In Vivo experiments) guidelines.

### Reparative dentin volume quantified by µCT analysis

Extracted maxillae after surgery were scanned by a µCT scanner (ScanXmate-L090T; Comscan, Tokyo, Japan) at 50 kV and 150 µA with a scanning resolution of 11.35 μm intervals in individual images. The volume of newly reparative dentin was quantified using TRI/3D/VIE-FCS software (Ratoc, Tokyo, Japan) (version 10.01.40.50-H-64).

### Immunohistochemical staining

DPSCs (8 × 10^4^ cells/dish) were cultured in 35-mm dishes for 1 day. After fixation with 4% PFA (Nacalai Tesque, Kyoto, Japan) and 0.5% dimethylsulfoxide (Nacalai Tesque) in phosphate-buffered saline (PBS) for 20 min, the cells were blocked with 2% BSA (Nacalai Tesque) in PBS for 1 h and then reacted with an anti-DA antibody (Abcam, Cambridge, UK, 1:1000) or normal rabbit IgG overnight at 4 °C. After cells were reacted with Alexa Fluor 568-conjugated goat anti-rabbit IgG (Invitrogen, Carlsbad, CA, USA, 1:1000) for 30 min at room temperature, nuclei were counterstained with a 4′,6-diamidino-2-phenylindole solution (Nacalai Tesque). Images of cells were obtained under the BZ-9000 microscope.

### Immunohistochemistry

After direct pulp capping, 7- or 8-week**-**old male Wister rats were perfused with 4% PFA in PBS under anesthesia. Maxillary bones were decalcified using Kalkitox (FUJIFILM Wako) at 4 °C for 3 days. They were embedded in paraffin and sectioned at 5 μm thicknesses. After deparaffinization, the tissues were incubated with 2% BSA in PBS for 1 h at room temperature and then reacted with an anti-Th antibody (Abcam, 1:500), anti-Nestin antibody (Invitrogen, 1:200–1:300), anti-DA antibody, (1:500), or normal rabbit IgG overnight at 4 °C. Then, after the tissues were incubated with biotinylated anti-rabbit IgG (Nichirei Biosciences, Tokyo, Japan) for 30 min at room temperature, they were reacted with an avidin-peroxidase conjugate (Nichirei Biosciences) for 30 min at room temperature. A simple stain DAB solution (Nichirei Biosciences) visualized positive staining. Nuclei were counterstained using Mayer’s hematoxylin solution (FUJIFILM Wako). Images of the tissues were obtained using the BZ-9000 microscope.

### Plasmid construction and lentivirus infection

Plasmid construction and lentivirus infection were performed as reported previously^46^. Briefly, to construct the lentiviral vector, human TH cDNA, which was synthesized in FASMAC, was inserted into the pTA2 vector and subcloned into CS2-CMV-MCS-IRES2-Bsd kindly provided by Dr. H. Miyoshi (RIKEN BioResource Center, Ibaraki, Japan). Then, the TH-inserted vector or Mock vector was cotransfected with packaging vectors pCAG-HIV-gp and pCMV-VSV-G-REV-rev into HEK293T cells using Lipofectamine LTX reagent to collect lentivirus particles. For lentivirus infection, DPSCs (2.5 × 10^4^ cells/well on 12-well plates) were cultured in 10% FBS/Dulbecco's Modified Eagle Medium (DMEM) containing the produced lentivirus particles and 10 mg/ml polybrene.

### Cell proliferation assay

We cultured DPSCs (1 × 10^3^ cells/well) on 48-well plates (Becton Dickinson Labware) for 24, 48, and 72 h in CM. The cell proliferation rate was measured using a Premix WST-1 Cell Proliferation Assay System (Takara Bio Inc.), following the manufacturer’s instructions.

### Western blotting

Proteins were collected from DPSCs using Pierce RIPA buffer (Invitrogen). The proteins were subjected to 10% sodium dodecyl sulfate‐polyacrylamide gel electrophoresis and subsequently transferred onto Immune-Blot PVDF membranes (Bio‐Rad Laboratories). After blocking with TBST containing 2% BSA or 5% dry skim milk (Yukijirushi, Tokyo, Japan) for 1 h at room temperature, the membranes were reacted with the anti-TH antibody (1:1000) or anti-β*-*actin antibody (1:1000) overnight at 4 °C. Then, the membranes were stained with biotinylated anti‐rabbit IgG (Nichirei Biosciences) or anti-mouse IgG (Nichirei Biosciences) and reacted with an avidin-peroxidase conjugate (1:2000, Sigma-Aldrich). Reactive bands were detected using the ECL select Western blotting detection system (GE Healthcare, Buckinghamshire, UK). Images were analyzed by Image Quant LAS 4000 (GE Healthcare) and ImageJ 1.53e (Java 1.8.0_172, http://imagej.nih.gov/ij).

### Flow cytometry

Surface antigen expression on DPSCs was investigated as described previously^47^. Briefly, after 2 × 10^4^ cells were reacted with CD29-PE, CD34-PE, CD45-PE, CD90-FITC, CD105-PE, CD146-FITC (eBioscience, San Diego, USA), and mouse IgG1 or IgG2a isotype control-PE (eBioscience), the samples were analyzed using an EC800 cell analyzer (Sony Biotechnology, Champaign, USA).

### Dopamine ELISA analysis

DPSCs were cultured in CM or DM (4 × 10^5^/well on 96-well plates). After 7 days of cultivation, each culture supernatant was collected. Additionally, cells expressing Mock or TH were cultured on 96-well plates (2 × 10^4^ /well) for 1 day. After stimulation with 2 mM CaCl_2_ for 30 min, the culture supernatant was collected. Collected supernatants were centrifuged at 1500 rpm for 10 min at 4 °C. The dopamine concentration in the supernatant was measured by a Dopamine ELISA kit (ImmuSmol, Pessac, France) following the manufacturer’s instructions.

### Small interfering RNA transfection

DPSCs were transfected with TH small interfering RNA (siRNA) (MISSION siRNA; SASI_Hs01_00200253, and SASI_Hs01_00061851, Sigma-Aldrich) or human control siRNA (MISSION siRNA Universal Negative Control #1, SIC-001-10; Sigma-Aldrich) using Lipofectamine RNA iMAX (Invitrogen) following the manufacturer’s protocols. In detail, DPSCs (2 × 10^4^ cells/well on 24-well plates) were cultured in Opti-MEM (Invitrogen) containing 10% FBS for 24 h. An siRNA-lipid complex, including 5 pmol siRNA and 1.5 µL Lipofectamine RNA iMAX in 50 µL Opti-MEM, was prepared. After incubation for 30 min at room temperature, the complex was added to the cells, and the cells were incubated for 24 h.

### Quantitative RT-PCR

PCR assays were performed using KAPA Express Extract (Kapa Biosystems, Woburn, MA, USA) in a Thermal Cycler Dice Real Time System (Takara Bio Inc.) in accordance with our previous report^29^. Primer sequences, annealing temperatures, cycle numbers, product sizes, and sequence IDs for *DMP-1*,* DSPP*, *NESTIN, TH*, and *β-actin* are shown in Supplementary Table 1. *β-actin* was used as an internal control.

### Semi-quantiative RT-PCR

Total RNA from DPSCs and SK-N-SH cells (RIKEN) was extracted using TRIzol reagent and reverse transcribed using an ExScript RT reagent Kit (Takara Bio Inc.) in a PCR Thermal Cycler Dice following the manufacturer’s instructions. PCR conditions were the same as those we reported previously^29^. Primer sequences, annealing temperatures, cycle numbers, product sizes, and sequence IDs for *DRD1*, *DRD2*, *DRD3, DRD4*, *DRD5*, and *GAPDH* are shown in Supplementary Table 2. *GAPDH* was used as an internal control. PCR products were separated by electrophoresis on 2% agarose gels (FUJIFILM Wako) containing ethidium bromide.

### Statistical analysis

Statistical analyses were performed using one-way ANOVA followed by Bonferroni’s test for four comparisons or the Student’s unpaired t-test with Easy R software (R version 4.0.3, https://www.jichi.ac.jp/saitama-sct/SaitamaHP.files/windowsEN.html)^48^. A probability value of *p* < 0.05 was considered as statistically significant.

## Date availability

The data analyzed during this study are included in this published article.

## Supplementary Information

Supplementary Information.

## References

[1] Sjogren U, Hagglund B, Sundqvist G, Wing K (1990). Factors affecting the long-term results of endodontic treatment. J. Endod..

[2] Suzuki S (2017). Number of non-vital teeth as indicator of tooth loss during 10-year maintenance: A retrospective study. Bull. Tokyo Dent. Coll..

[3] Kawahara H, Inoue M, Okura K, Oshima M, Matsuka Y (2021). Risk factors for tooth loss in patients with ≥25 remaining teeth undergoing mid-long-term maintenance: A retrospective study. Int. J. Environ. Res. Public Health.

[4] Tziafas D (2019). Characterization of odontoblast-like cell phenotype and reparative dentin formation in vivo: A comprehensive literature review. J. Endod..

[5] Dimitrova-Nakov S, Baudry A, Harichane Y, Kellermann O, Goldberg M (2014). Pulp stem cells: Implication in reparative dentin formation. J. Endod..

[6] Zhao L (2021). Odontoblast death drives cell-rich zone-derived dental tissue regeneration. Bone.

[7] Kawashima N, Okiji T (2016). Odontoblasts: Specialized hard-tissue-forming cells in the dentin-pulp complex. Congenit. Anom..

[8] Hilton TJ, Ferracane JL, Mancl L (2013). Comparison of CaOH with MTA for direct pulp capping: A PBRN randomized clinical trial. J. Dent. Res..

[9] Zhu C, Ju B, Ni R (2015). Clinical outcome of direct pulp capping with MTA or calcium hydroxide: A systematic review and meta-analysis. Int. J. Clin. Exp. Med..

[10] Komabayashi T, Zhu Q, Eberhart R, Imai Y (2016). Current status of direct pulp-capping materials for permanent teeth. Dent. Mater. J..

[11] Cushley S (2021). Efficacy of direct pulp capping for management of cariously exposed pulps in permanent teeth: A systematic review and meta-analysis. Int. Endod. J..

[12] Rashid F (2003). The effect of extracellular calcium ion on gene expression of bone-related proteins in human pulp cells. J. Endod..

[13] Smith AJ, Murray PE, Sloan AJ, Matthews JB, Zhao S (2001). Trans-dentinal stimulation of tertiary dentinogenesis. Adv. Dent. Res..

[14] Cox CF, Sübay RK, Ostro E, Suzuki S, Suzuki SH (1996). Tunnel defects in dentin bridges: Their formation following direct pulp capping. Oper Dent..

[15] Nagatsu T, Levitt M, Udenfriend S (1964). Tyrosine hydroxylase. The initial step in norepinephrine biosynthesis. J. Biol. Chem..

[16] Miyajima K (2021). Tyrosine hydroxylase conditional KO mice reveal peripheral tissue-dependent differences in dopamine biosynthetic pathways. J. Biol. Chem..

[17] Thomas Broome S (2020). Dopamine: An immune transmitter. Neural Regen. Res..

[18] Lee DJ (2015). Dopaminergic effects on in vitro osteogenesis. Bone Res..

[19] Lee DJ, Lee YT, Zou R, Daniel R, Ko CC (2017). Polydopamine-laced biomimetic material stimulation of bone marrow derived mesenchymal stem cells to promote osteogenic effects. Sci. Rep..

[20] Fujino S (2020). Expression and function of dopamine in odontoblasts. J. Cell Physiol..

[21] Quispe-Salcedo A, Ida-Yonemochi H, Nakatomi M, Ohshima H (2012). Expression patterns of nestin and dentin sialoprotein during dentinogenesis in mice. Biomed. Res..

[22] Takeuchi R (2020). Immunohistochemistry and gene expression of GLUT1, RUNX2 and MTOR in reparative dentinogenesis. Oral Dis..

[23] Schmidt U, Pilgrim C, Beyer C (1998). Differentiative effects of dopamine on striatal neurons involve stimulation of the cAMP/PKA pathway. Mol. Cell Neurosci..

[24] López-Sánchez C (2010). Tyrosine hydroxylase is expressed during early heart development and is required for cardiac chamber formation. Cardiovasc. Res..

[25] Vázquez P, Robles AM, de Pablo F, Hernández-Sánchez C (2014). Non-neural tyrosine hydroxylase, via modulation of endocrine pancreatic precursors, is required for normal development of beta cells in the mouse pancreas. Diabetologia.

[26] Schwendich E (2022). Modulation of dopamine receptors on osteoblasts as a possible therapeutic strategy for inducing bone formation in arthritis. Cells.

[27] Tada H (2010). Elevated extracellular calcium increases expression of bone morphogenetic protein-2 gene via a calcium channel and ERK pathway in human dental pulp cells. Biochem. Biophys. Res. Commun..

[28] da Rosa WLO, Piva E, da Silva AF (2018). Disclosing the physiology of pulp tissue for vital pulp therapy. Int. Endod. J..

[29] Mizumachi H (2017). Calcium-sensing receptor-ERK signaling promotes odontoblastic differentiation of human dental pulp cells. Bone.

[30] Huang HW, Zuo C, Chen X, Peng YP, Qiu YH (2016). Effect of tyrosine hydroxylase overexpression in lymphocytes on the differentiation and function of T helper cells. Int. J. Mol. Med..

[31] Wang CX (2020). Dopamine D1 receptor-mediated activation of the ERK signaling pathway is involved in the osteogenic differentiation of bone mesenchymal stem cells. Stem Cell Res. Ther..

[32] Zhu J (2022). Activation of dopamine receptor D1 promotes osteogenic differentiation and reduces glucocorticoid-induced bone loss by upregulating the ERK1/2 signaling pathway. Mol. Med..

[33] Lu Z (2020). Electrospun highly porous poly(L-lactic acid)-dopamine-SiO_2_ fibrous membrane for bone regeneration. Mater. Sci. Eng. C Mater. Biol. Appl..

[34] Wu J (2019). Functionalization of silk fibroin electrospun scaffolds via BMSC affinity peptide grafting through oxidative self-polymerization of dopamine for bone regeneration. ACS Appl. Mater. Interfaces.

[35] Baudry A (2015). Essential roles of dopamine and serotonin in tooth repair: Functional interplay between odontogenic stem cells and platelets. Stem Cells.

[36] Ipposhi K (2021). Secreted frizzled-related protein 1 promotes odontoblastic differentiation and reparative dentin formation in dental pulp cells. Cells.

[37] Yoshida S (2016). Semaphorin 3A induces odontoblastic phenotype in dental pulp stem cells. J. Dent. Res..

[38] Levite M (2016). Dopamine and T cells: Dopamine receptors and potent effects on T cells, dopamine production in T cells, and abnormalities in the dopaminergic system in T cells in autoimmune, neurological and psychiatric diseases. Acta Physiol..

[39] Komichi S (2019). Protein S100–A7 derived from digested dentin is a critical molecule for dentin pulp regeneration. Cells.

[40] Matsui M, Kobayashi T, Tsutsui TW (2018). CD146 positive human dental pulp stem cells promote regeneration of dentin/pulp-like structures. Hum. Cell.

[41] Ma L (2021). CD146 controls the quality of clinical grade mesenchymal stem cells from human dental pulp. Stem Cell Res. Ther..

[42] An S (2019). The emerging role of extracellular Ca^2+^ in osteo/odontogenic differentiation and the involvement of intracellular Ca^2+^ signaling: From osteoblastic cells to dental pulp cells and odontoblasts. J. Cell Physiol..

[43] Nozu A (2018). Senescence and odontoblastic differentiation of dental pulp cells. J. Cell Physiol..

[44] Murakami S (2017). Dose effects of beta-tricalcium phosphate nanoparticles on biocompatibility and bone conductive ability of three-dimensional collagen scaffolds. Dent. Mater. J..

[45] Gorseta K, Glavina D (2017). Thermo-cured glass ionomer cements in restorative dentistry. J. Istanb Univ. Fac. Dent..

[46] Fujii S (2019). Wnt/β-catenin signaling, which is activated in odontomas, reduces Sema3A expression to regulate odontogenic epithelial cell proliferation and tooth germ development. Sci. Rep..

[47] Wada N, Menicanin D, Shi S, Bartold PM, Gronthos S (2009). Immunomodulatory properties of human periodontal ligament stem cells. J. Cell Physiol..

[48] Kanda Y (2013). Investigation of the freely available easy-to-use software 'EZR' for medical statistics. Bone Marrow Transpl..

