# Supplementary material for: Dopamine is involved in reparative dentin formation through odontoblastic differentiation of dental pulp stem cells
Source: Sci Rep. 2023 Apr 6;13:5668. doi: 10.1038/s41598-023-32126-1 (PMC10079685; doi:10.1038/s41598-023-32126-1)
Supplement: Supplementary file 1 — Supplementary Information. [file 41598_2023_32126_MOESM1_ESM.docx]

**Supplementary Material For**

Dopamine is involved in reparative dentin formation through odontoblastic differentiation of dental pulp stem cells

**Authors**

Shoko Fujino^1^, *Sayuri Hamano^1,2^, Atsushi Tomokiyo^3^, Risa Sugiura^1^, Daiki Yamashita^1^, Daigaku Hasegawa^3^, Hideki Sugii^1^, Shinsuke Fujii^4^, Tomohiro Itoyama^1^, Hirofumi Miyaji^5^, Hidefumi Maeda^1,3^

^1^Department of Endodontology and Operative Dentistry, Kyushu University, ^2^OBT Research Center, Kyushu University, ^3^Department of Endodontology, Kyushu University Hospital, ^4^Laboratory of Oral Pathology, Division of Maxillofacial Diagnostic and Surgical Sciences, Faculty of Dental Science, Kyushu University, ^5^Department of Periodontology and Endodontology, Faculty of Dental Medicine, Hokkaido University.

***Corresponding author:** Sayuri Hamano, DDS, PhD

Department of Endodontology and Operative Dentistry,

Faculty of Dental Science, Kyushu University

3-1-1 Maidashi Fukuoka 812-8582, Japan.

Phone: +81-92-642-6432

Fax: +81-92-642-6366

E-mail: shamano@dent.kyushu-u.ac.jp

**
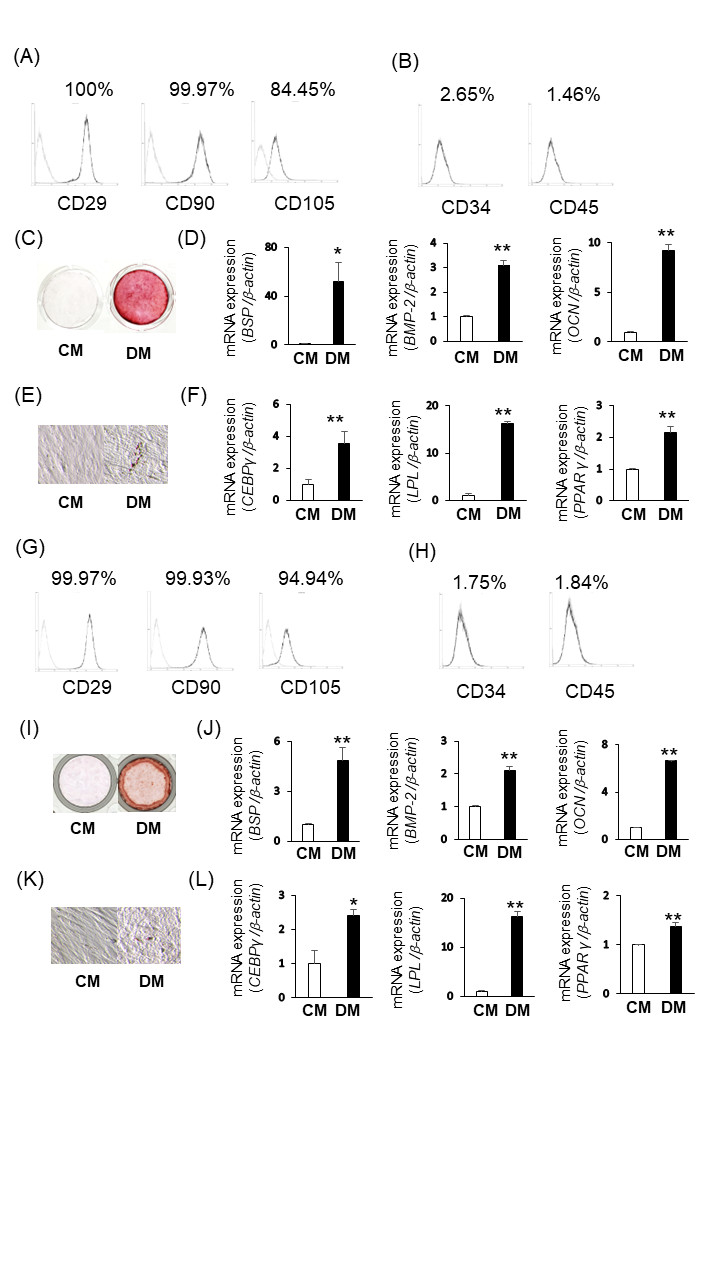
**

**Supplementary Figure 1. Characterization of DPSCs-3U and -5C.** Characteristics of stem cells in DPSCs-3U (A-F) and -5C (G-L) were examined. Expression of MSC markers, CD29, CD90, and CD105, (A, G) and hematopoietic stem cell markers, CD34 and CD45 (B, H) of DPSCs-3U and -5C were investigated by flow cytometry analysis. DPSCs -3U and -5C were cultured in osteogenic (C, D, I, J), and adipogenic (E, F, K, L) medium for 4weeks. (C, I) Calcium deposits were investigated by Alizarin Red S staining in DPSCs-3U (C) and -5C (I). (D, J) Gene expression of BSP, BMP-2, and OCN in DPSCs-3U (D) and -5C (J) was examined by quantitative RT-PCR. (E, K) Intrafat drop was detected by Oil Red O staining in DPSCs-3U (E) and -5C (K). (F, L) Gene expression of CEBPg, LPL, and PPARg in DPSCs-3U (F) and -5C (L) was examined by quantitative RT-PCR. β-actin was used an internal control. Statistical analysis was performed using the Student’s unpaired t-test with Easy R software. n=3, **P<0.01, *P<0.05.


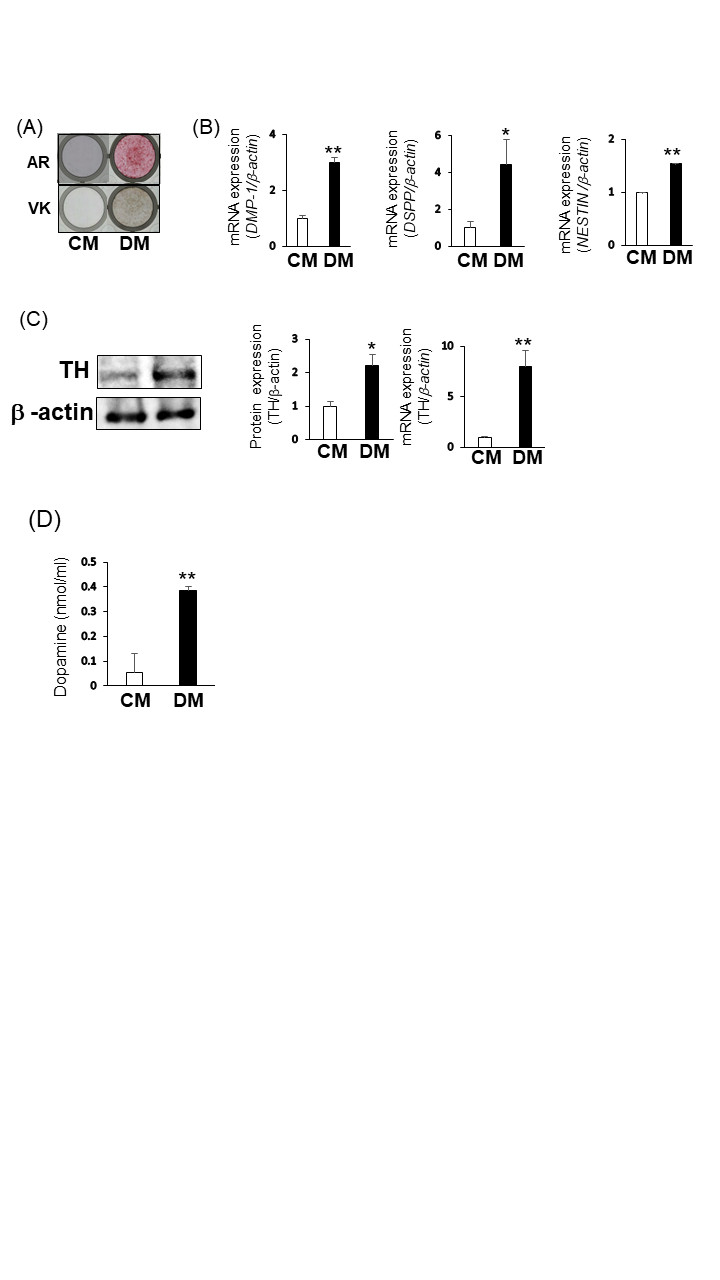


**Supplementary Figure 2. TH and DA expression during odontoblastic differentiation of DPSCs-5C.** (A-C) DPSCs were cultured in CM or DM. (A) After 7 days of cultivation, calcium deposits were analyzed by Alizarin Red S staining and von Kossa staining. (B) After 3days of cultivation, gene expression of DMP-1, DSPP, NESTIN was examined by quantitative RT-PCR. (C) Gene and protein expression of TH was investigated by quantitative RT-PCR and Western blotting. The bands were quantified using ImageJ. Original blots are presented in Supplementary Information 8. (D) DA concentration of the culture supernatant of DPSCs was analyzed by ELISA. Statistical analysis was performed using the Student’s unpaired t-test with Easy R software. n=3, **P<0.01, *P<0.05.


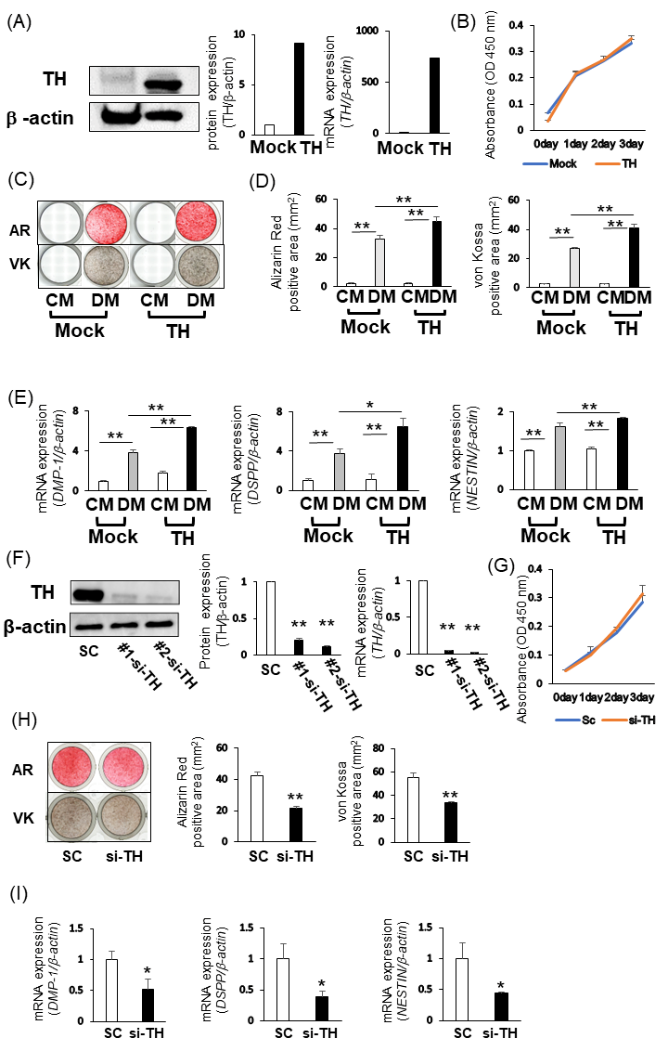


**Supplementary Figure 3. Effects of TH overexpression on the odontoblastic differentiation of DPSCs-5C.** (A-E) We cultured Mock- and TH-DPSCs in CM or DM. (A) Gene and protein expression of TH were investigated by quantitative RT-PCR and Western blotting. The bands were quantified using ImageJ. (B) Proliferation of Mock-DPSCs or TH-DPSCs was examined by WST-1 assay. (C) Alizarin Red S staining and von Kossa staining of Mock-DPSCs or TH-DPSCs cultured in CM or DM. (D) Positive area of Alizarin Red S staining and von Kossa staining was quantified using a Keyence BZ-9000 microscope with BZ-H4M/BZ-H4C/BZ-H4CM software. (E) Gene expression of DMP-1, DSPP, and NESTIN was investigated by quantitative RT-PCR. (F-I) TH-DPSCs was transfected with control siRNA or TH siRNA. (F) Gene and protein expression of TH were confirmed by quantitative RT-PCR and Western blotting. The bands were quantified using ImageJ. (G) Proliferation of TH-DPSCs transfected with control siRNA or TH siRNA was examined by WST-1 assay. (H) Alizarin Red S staining and von Kossa staining of TH-DPSCs transfected with control siRNA or TH siRNA cultured in DM. The positive areas were quantified by Keyence BZ-9000 microscope with BZ-H4M/BZ-H4C/BZ-H4CM software. (I) Gene expression of DMP-1, DSPP and NESTIN was examined by quantitative RT-PCR. Statistical analysis was performed using the Student’s unpaired t-test or one-way ANOVA followed by Bonferroni’s test with Easy R software. n=3, **P<0.01, *P<0.05. Original blots are presented in Supplementary Information 8.


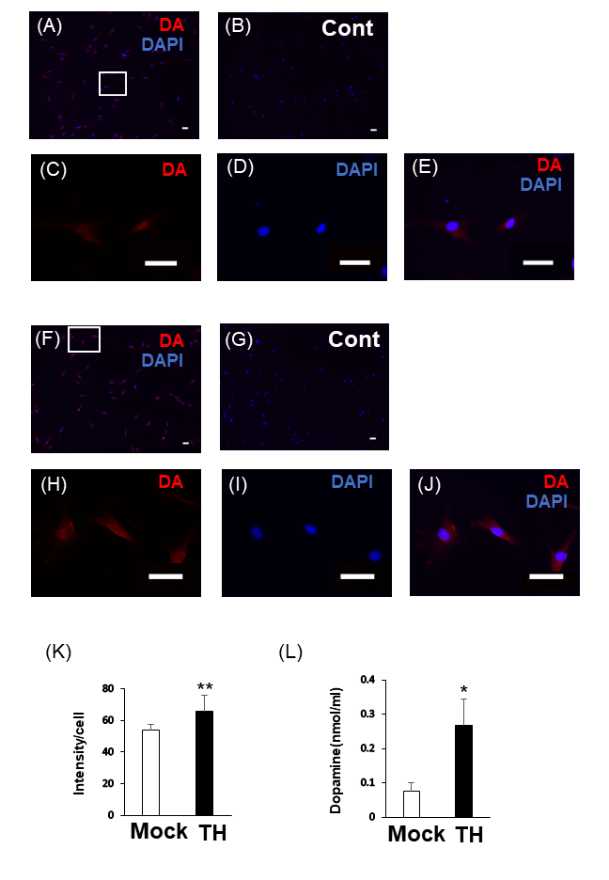


**Supplementary Figure 4. Dopamine production in Mock- or TH-DPSCs (5C).** (A-J) Immunofluorescence staining of Mock-DPSCs or TH-DPSCs with anti-DA antibody (red) was performed. (B, G) Rabbit control IgG (cIgG) was used as a negative control. (C-E) Magnified images of the white boxed areas in (A). (H-J) Magnified images of the white boxed areas in (F). Nuclei were stained with DAPI. Bars=50 μm. (K) Intensity of immunofluorescence staining of DA were quantified using a Keyence BZ-9000 microscope with BZ-H4M/BZ-H4C/BZ-H4CM software. (L) Mock-DPSCs or TH-DPSCs were cultured in DM for 30 min. The concentration of DA of each culture supernatant was measured by ELISA analysis. Statistical analysis was performed using the Student’s unpaired t-test with Easy R software. n=3, **P<0.01, *P<0.05.

**
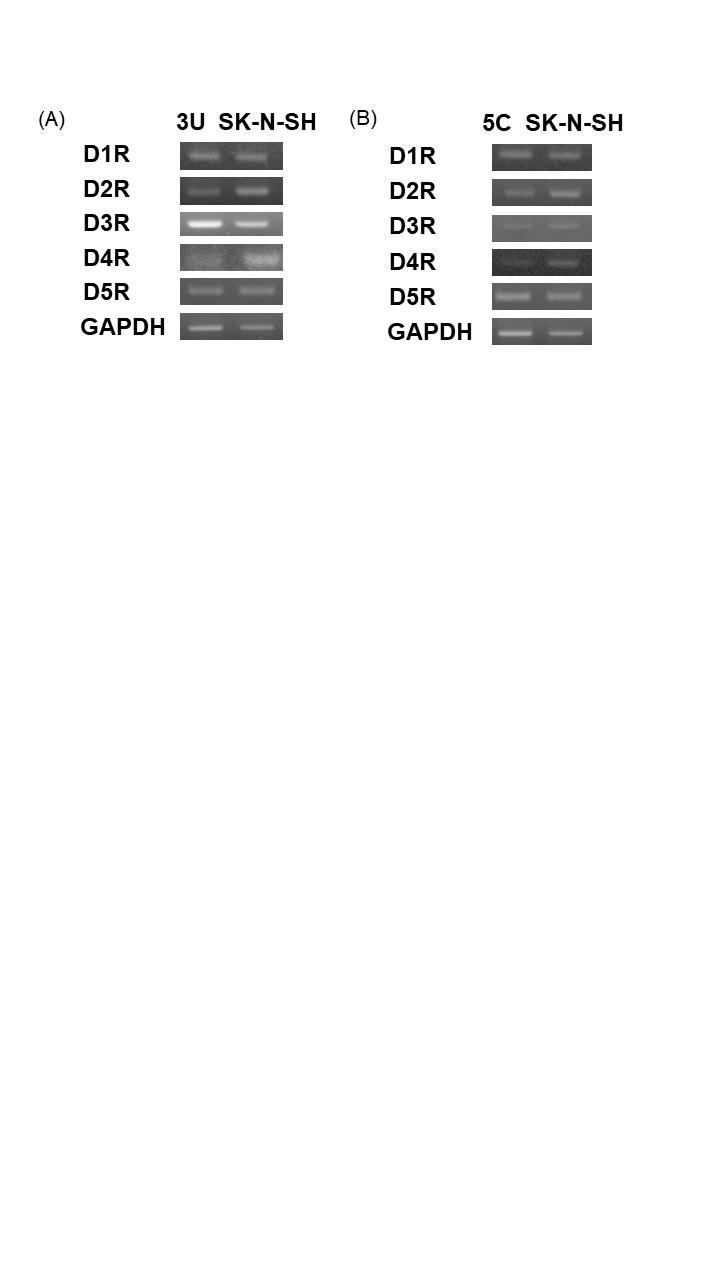
**

**Supplement Figure 5. Expression of *dopamine receptors* in DPSCs-3U and 5C.**

(A, B) Gene expression of dopamine receptors (D1R, D2R, D3R, D4R, and D5R) of U- (A) and 5C- (B) DPSCs was examined by semi-quantitative RT-PCR. Gene of SK-N-SH was used as a positive control. Original gels are presented in Supplementary Information 8.


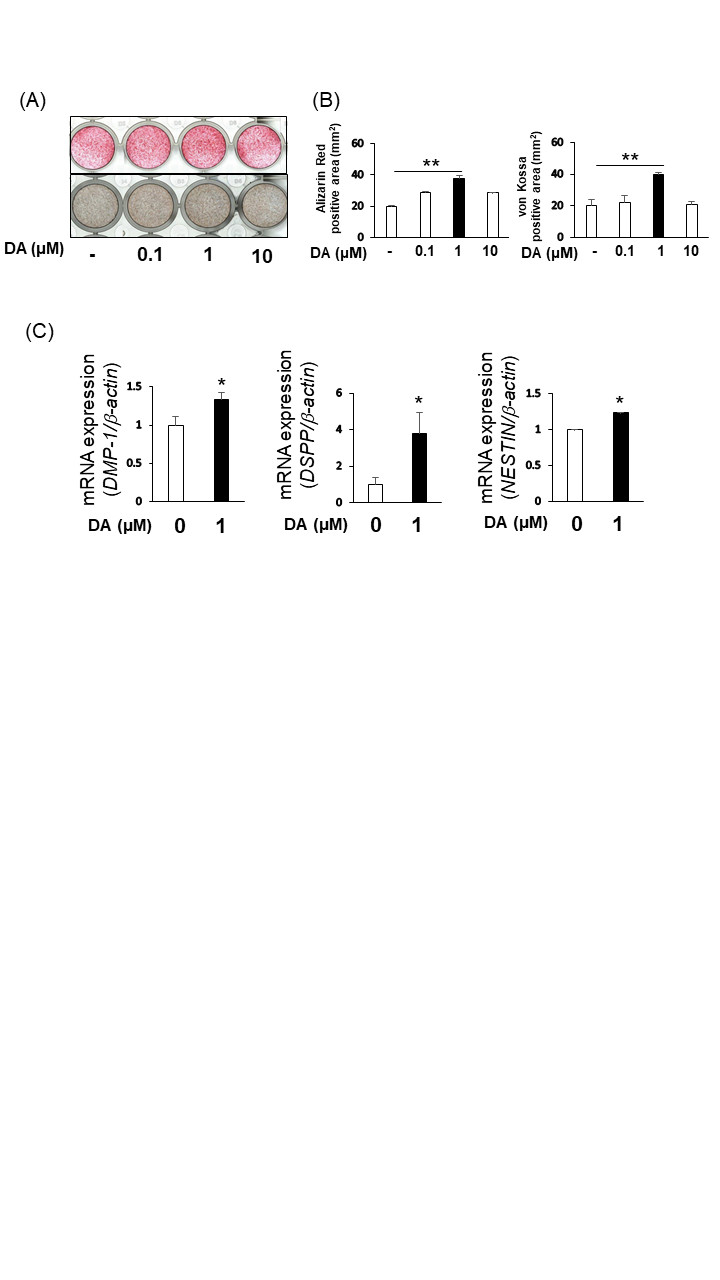


**Supplement Figure 6.** **Effects of DA on the odontoblastic differentiation of DPSCs-5C.** (A-C) DPSCs were cultured in DM containing without or with dopamine (0.1, 1, and 10 mM). (A) After 7 days of cultivation, calcium deposits were analyzed by Alizarin Red S staining and von Kossa staining. (B) These images were quantified using a Keyence BZ-9000 microscope with BZ-H4M/BZ-H4C/BZ-H4CM software. (C) After 3 days of cultivation, the gene expression of DMP-1, DSPP and NESTIN were investigated by quantitative RT-PCR. Statistical analysis was performed using the Student’s unpaired t-test or one-way ANOVA followed by Bonferroni’s test with Easy R software. n=3, **P<0.01, *P<0.05.


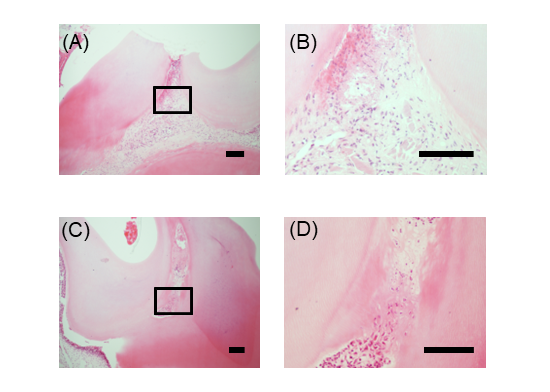


**Supplementary Figure 7.** **Direct pulp capping treatment with DA (10^-2^ and 10^-1^ M).** HE staining of the maxillary first molars at day 21 after direct pulp capping treatment with nano β-TCP/collagen scaffold containing 10^-2^ M (A, B) or 10^-1^ M (C, D) DA. (B, D) Magnified images of the black boxed areas in (A, C). Bars=100 μm.


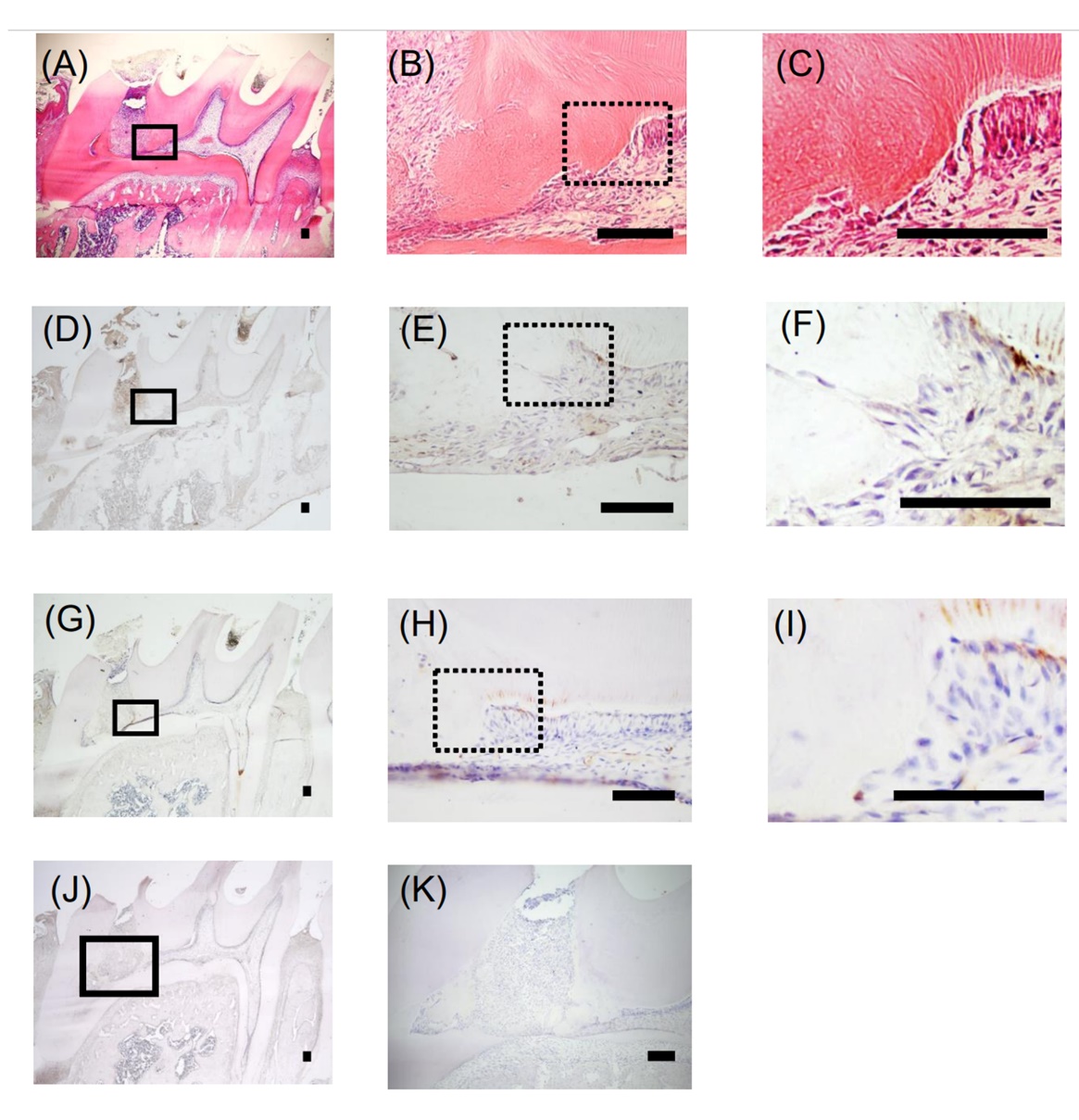


**Supplement Figure 8.** **Direct pulp capping treatment with DW (Control group)**. The surface of exposed pulp was capped with nano β-TCP/collagen scaffolds containing DW. (A-C) HE staining of the maxillary first molars at day 21 after direct pulp capping treatment with DW. (D-K) Immunohistochemical staining of the maxillary first molars in Control group with anti-Nestin antibody (D-F) and anti- Th antibody (G-I). (B, E, H, K) Magnified images of the black boxed area in (A, D, G, J). (C, F, I) Magnified images of the black dotted boxed area in (B, E, H). (J, K) Rabbit control IgG was used as a negative control. Nuclei were stained with hematoxylin. Bars=100 µm.


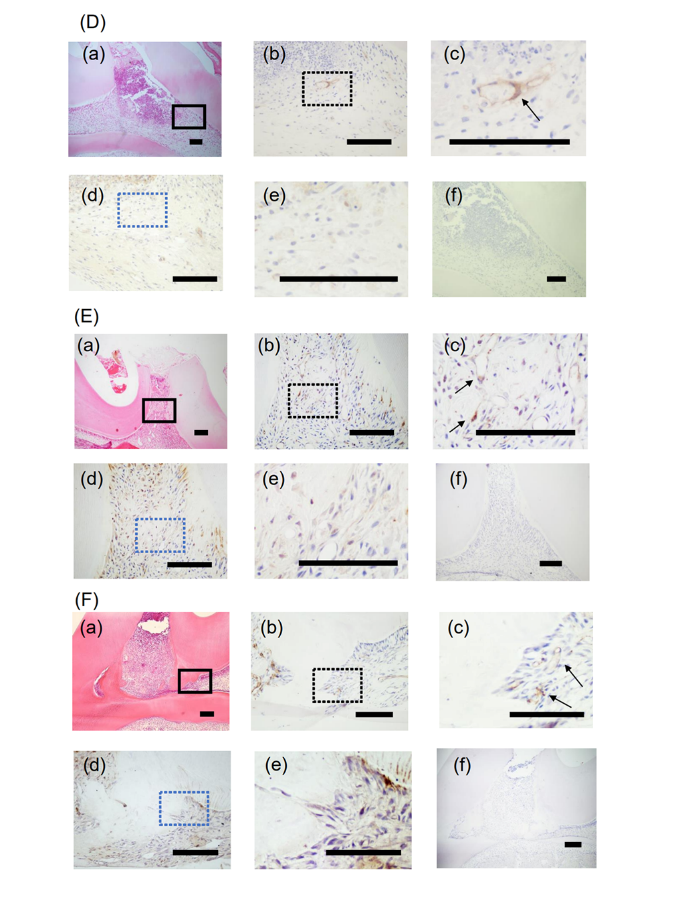

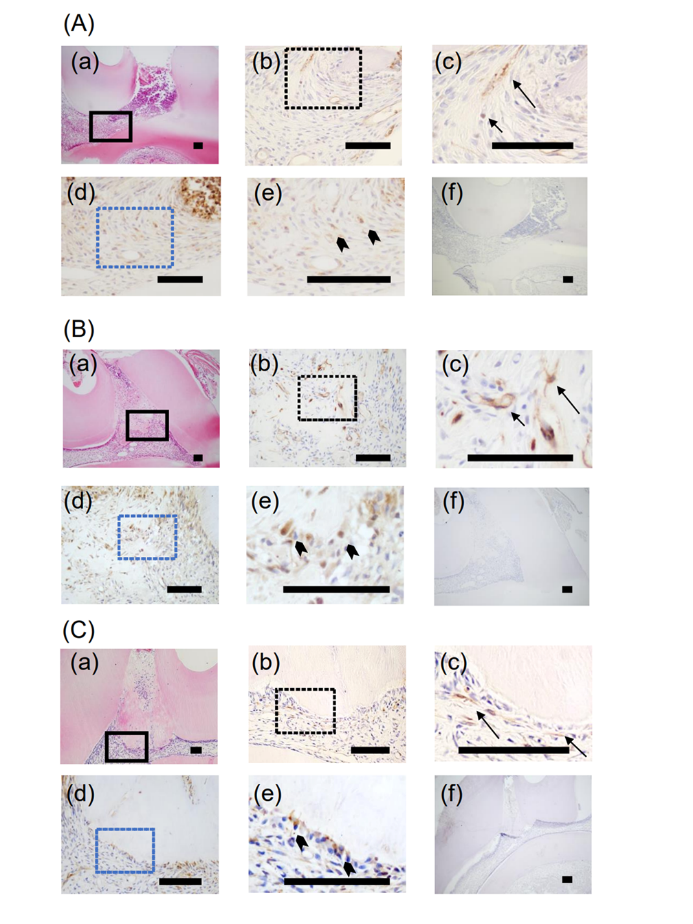


**Supplementary Figure 9. Expression of CD146 and Nestin in pulp tissues in rat direct pulp capping model.** The surface of exposed pulp was capped with nano β-TCP/collagen scaffold containing DA (A-C) or DW (D-F). Images of maxillary first molars at day 3 [A(a-f), D(a-f)], day 7 [B(a-f), E(a-f)], and day 21 [C(a-f), F(a-f))] after treatment. HE staining of the maxillary first molars [A-F(a)]. Immunohistochemical staining of the maxillary first molars with anti-CD146 antibody [A-F(b, c)] and anti- Nestin antibody [A-F(d, e)]. [A-F(c)] Magnified images of the black boxed areas in [A-F(b)]. [A-F(e)] Magnified images of the blue dotted boxed areas in [A-F(d)]. [A-F(f)] Rabbit control IgG was used as a negative control. Nuclei were stained with hematoxylin. Bars=100 μm. Arrows; CD146- positive cells. Arrowheads; Nestin- positive cells.


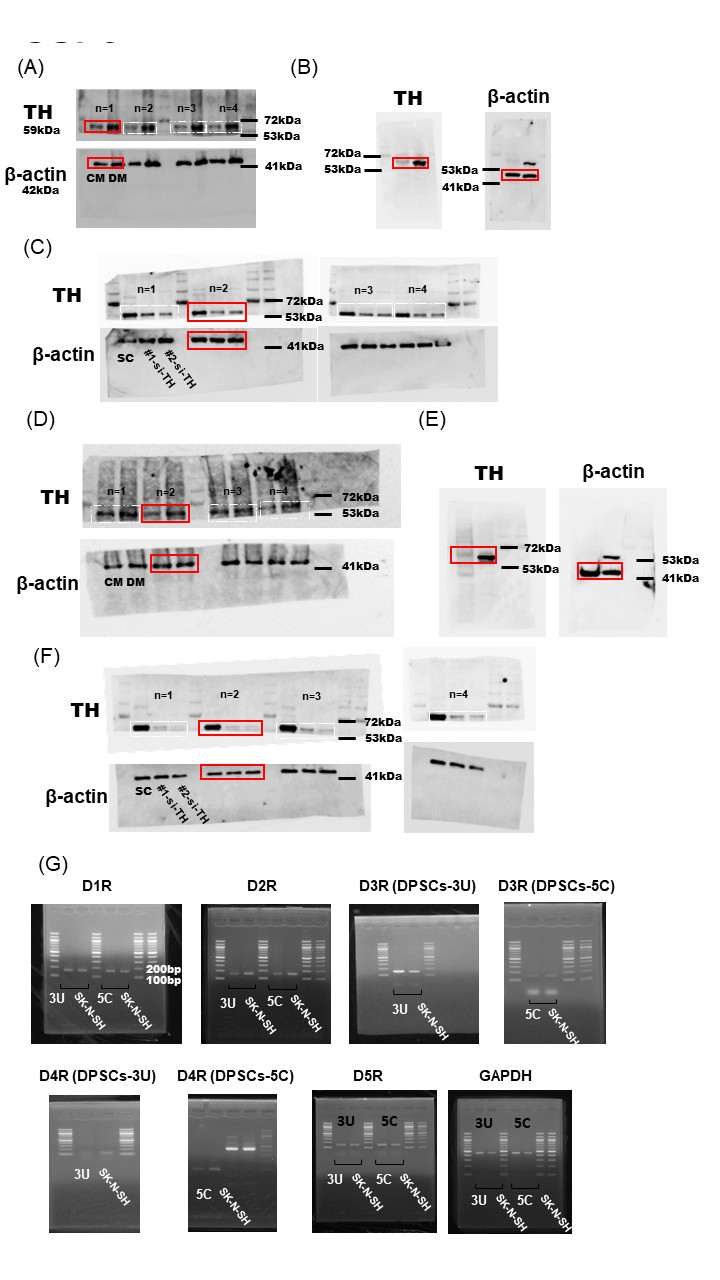


**Supplementary Figure 10. Full scan images of immunoblots and electorophoresis.** (A-F) The full western bolt images in Fig. 2C, Fig. 3A, Fig. 3F, Sup. 2C, Sup. 3A, and, Sup. 3F. (G) The full electrophoresis images in Supplementary Fig. 5.

| Target gene  (abbreviation) | Forward (top) and reverse (bottom)  primer sequences | Size of amplified products (bp) | Annealing temperature(℃) | Cycles | Sequence ID |
| --- | --- | --- | --- | --- | --- |
| DMP-1 | CCCTTGGAGAGCAGTGAGTC  CTCCTTTTCCTGTGCTCCTG | 165 | 60 | 40 | NM_001079911 |
| DSPP | ATATTGAGGGCTGGAATGGGGA  TTTGTGGCTCCAGCATTGTCA | 136 | 60 | 40 | NM_014208 |
| NESTIN | TGGCCACGTACAGGACCCTCC  AGATCCAAGACGCCGGCCCT | 143 | 60 | 40 | [NM_006617](https://www.ncbi.nlm.nih.gov/entrez/viewer.fcgi?db=nucleotide&id=38176299) |
| TH | ATGCCGGTACTGGTTCTTCC  TGCCGAAAGGAAATGGGTCA | 90 | 60 | 40 | [NM_001346091](https://www.ncbi.nlm.nih.gov/entrez/viewer.fcgi?db=nucleotide&id=1069561758) |
| β-actin | ATTGCCGACAGGATGCAGA  GAGTACTTGCGCTCAGGAGGA | 89 | 60 | 40 | NM_001101 |

**Supplementary Table 1.** Primer sequences, product size, annealing temperatures, cycle numbers, and sequence IDs for quantitative RT-PCR.

| Target gene  (abbreviation) | Forward (top) and reverse (bottom)  primer sequences | Size of amplified products (bp) | Annealing temperature(℃) | Cycles | Sequence ID |
| --- | --- | --- | --- | --- | --- |
| DRD1 | CTCCGTTTCCAAATACATTCCA  CACTGTTGATTCTTTGCCCT | 169 | 54 | 35 | NM_000794 |
| DRD2 | AGCATCGACAGGTACACAG  CTCGTTCTGGTCTGCGT | 159 | 54 | 35 | NM_000795 |
| DRD3 | TCTGTGCCATCAGCATAGACAGG  TAAGCCAAACAGAAGAGGGCAGG | 143 | 58 | 40 | NM_033660 |
| DRD4 | CCGCTCTTCGTCTACTC  ACAGGTTGAAGATGGAGG | 114 | 54 | 45 | NM_000797 |
| DRD5 | CTCATCTCCTACAACCAAGAC  TGATAGATCTGGAACATGCGA | 148 | 54 | 35 | NM_000798 |
| GAPDH | ACCACAGTCCATGCCATCCAC  TCCACCACCCTGTTGCTGTA | 452 | 60 | 20 | NM_001256799 |

**Supplementary Table 2.** Primer sequences, product size, annealing temperatures, cycle numbers, and sequence IDs for semi-quantitative RT-PCR.
